# Structure of the replication regulator Sap1 reveals functionally important interfaces

**DOI:** 10.1038/s41598-018-29198-9

**Published:** 2018-07-19

**Authors:** Maria M. Jørgensen, Babatunde Ekundayo, Mikel Zaratiegui, Karen Skriver, Geneviève Thon, Thomas Schalch

**Affiliations:** 1 Department of Biology, University of Copenhagen, Copenhagen, Denmark; 2 Department of Molecular Biology, Science III, Institute of Genetics and Genomics of Geneva (iGE3), University of Geneva, CH-1211 Geneva 4, Switzerland; 3 Department of Molecular Biology and Biochemistry, Rutgers University, Piscataway, USA; 4 Leicester Institute for Structural and Chemical Biology, Department of Molecular and Cell Biology, University of Leicester, Leicester, LE1 9HN UK

## Abstract

The mechanism by which specific protein-DNA complexes induce programmed replication fork stalling in the eukaryotic genome remains poorly understood. In order to shed light on this process we carried out structural investigations on the essential fission yeast protein Sap1. Sap1 was identified as a protein involved in mating-type switching in *Schizosaccharomyces pombe*, and has been shown to be involved in programmed replication fork stalling. Interestingly, Sap1 assumes two different DNA binding modes. At the mating-type locus dimers of Sap1 bind the SAS1 sequence in a head-to-head arrangement, while they bind to replication fork blocking sites at rDNA and Tf2 transposons in a head-to-tail mode. In this study, we have solved the crystal structure of the Sap1 DNA binding domain and we observe that Sap1 molecules interact in the crystal using a head-to-tail arrangement that is compatible with DNA binding. We find that Sap1 mutations which alleviate replication-fork blockage at Tf2 transposons in CENP-B mutants map to the head-to-tail interface. Furthermore, several other mutations introduced in this interface are found to be lethal. Our data suggests that essential functions of Sap1 depend on its head-to-tail oligomerization.

## Introduction

As replication forks progress along the genome, they face many challenges including DNA damage lesions, strand breaks and DNA binding proteins which may cause the replication fork to pause or stall. In the event that these hurdles are not properly repaired or processed, stalled forks could collapse leading to genomic instability^1^.

Nonetheless, programmed fork stalling occurs at specific sites in genomes mediated by the interaction of these sites with transacting barrier proteins^2^. A well-studied example of this mechanism is the Tus-Ter complex in *E. coli*^3^. *E. coli* is able to minimize collisions between DNA and RNA polymerases as its circular DNA is being replicated by oppositely oriented forks with fork barrier sites in the terminus region of its genome, such that the most active genes are replicated and transcribed in the same direction^4^. The DNA replication termination sites known as Ter sites are bound by a protein called Tus. As replication forks encounter the Tus-Ter complex they displace Tus from Ter sites when approaching from the permissive face of the complex, however in the opposite orientation replication forks encounter the non-permissive face of the complex and are therefore halted until replication terminates from the other side. Fork blockage is mediated by a ‘molecular mouse trap mechanism’ in which a cytosine in the Ter site flips into a specific pocket on Tus during strand separation as the oncoming helicases approach the non-permissive face of the complex^5^. This locking mechanism was recently discussed in the light of single molecule studies^3^. Unlike the Tus-Ter locking mechanism described in *E. coli*, the mechanisms underlying replication fork stalling by barrier proteins bound to specific sites in eukaryotic genomes remain unknown.

**Figure 1:**
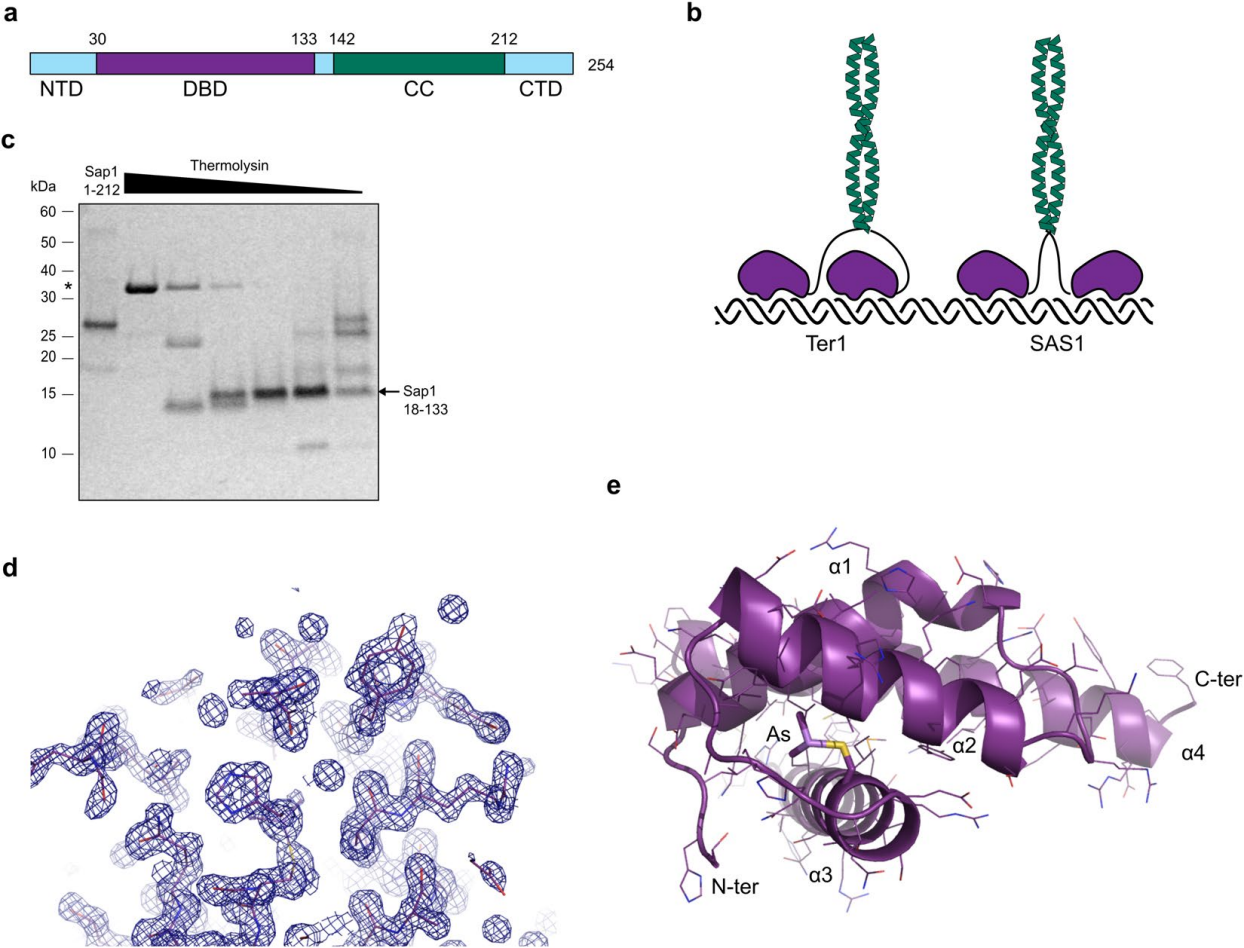
Crystallization and structure determination of the Sap1 DNA binding domain. (**a**) Domain diagram of Sap1 with N-terminal domain (NTD), DNA binding domain (DBD), coiled-coil (CC) and C-terminal domain. (**b**) Illustration of binding modes proposed for Sap1 dimers binding to Ter1 (direct repeat) or SAS1 (inverted repeats). (**c**) Limited proteolysis using thermolysin on Sap1 protein comprising residues 1–212. Thermolysin is marked by asterisk. (**d**) Electron density after refinement at σ = 1.5 for a randomly selected region of Sap1. (**e**) Cartoon representation of the Sap1 structure with the arsenic adduct dimethylarsinoyl to Cys81 shown in stick representation.

In yeast, fork stalling at specific sites is required for faithful replication of repetitive sequences and factors involved in this process are essential for cell growth^6^. The fission yeast essential protein Sap1 is an abundant DNA binding protein bearing a DNA binding domain and a long coiled-coil that induces homodimerization^7^ (Fig. 1a). Sap1 was first described as a trans-acting factor of the SAS1 element at the mating-type locus that is required for mating-type switching^8^. However, it has also been shown to bind Ter1 sites at the rDNA repeats where it stalls replication fork progression^9,10^. In contrast, Sap1 does not stall replication fork progression at SAS1. Sequence comparison between Ter1 and SAS1 reveals that the Sap1 binding motifs occur as inverted repeats at SAS1 and as direct tandem repeats at Ter1 (Fig. 1b)^11^. This implies that a Sap1 dimer is able to interact with its binding sites in two modes in which the same face of the complex is exposed to the oncoming replication fork when bound to SAS1 and two different faces of the protein are exposed when bound to Ter1, suggesting different intramolecular interactions between Sap1 monomers when bound to Ter1 or SAS1. These differing intramolecular interactions could have a role in the mechanism of replication fork stalling by Sap1.

It has also been shown that Sap1 guides the integration of transposons to promoter regions of genes, a process which requires both its fork stalling ability and its binding in a direct repeat orientation^12^. Furthermore, a missense mutation in Sap1 suppresses the lethal phenotype of CENP-B deletions^13^. CENP-B proteins are required for progression of the replication fork through long terminal repeats of retrotransposons, and the Sap1-c mutation alleviates the replication fork blockage caused by CENP-B deletion. Even though Sap1 biology has been extensively studied, little is known about the mechanistic aspects of how Sap1 elicits its functions at the molecular level. In order to gain more insight into these processes we carried out structural studies on Sap1. Here we present the crystal structure of the Sap1 DNA binding domain which allows us to model Sap1 bound to DNA in its proposed replication fork stalling orientation based on structural homology searches and biochemical data.

In our crystal structure we have identified a dimerization interface between Sap1 monomers that we predict to be engaged in the direct repeat binding mode. Interestingly, mutations in Sap1 which rescue the CENP-B deletion phenotype lie at or near this dimerization interface. Furthermore, we show by mutational analysis that this interface is essential for Sap1 function and fission yeast viability.

## Results

### Structure of the Sap1 DNA binding domain solved by arsenic anomalous scattering

In order to provide a structural basis for understanding replication fork stalling by Sap1 we set out to crystallize Sap1. Initial trials were performed with the dimeric form of Sap1 comprising residues 25–150 and we promptly obtained crystals. However, the crystals were difficult to reproduce, and we hypothesized that a degraded form of the protein might be forming the crystals. Indeed, limited proteolysis yielded a very stable fragment that we mapped to residues 18–133 of Sap1 (Fig. 1c), corresponding to the DNA binding domain^14^. We expressed residues 18–133 and readily obtained crystals that diffracted to 1.4 Å resolution (Table 1). Since molecular replacement was not an option due to lack of sequence homologs, we attempted to solve the structure by anomalous diffraction using selenomethionine labeling. Unexpectedly however, the structure solution was greatly facilitated by anomalous scattering originating from an arsenic adduct (dimethylarsinoyl) to Cys81, formed due to the cacodylate buffer used in the crystallization solution used in the PACT screen (25% w/v PEG 1500, 0.1 M PCTP pH 7.0 (propionic acid, cacodylate and bis-tris propane)) (Figs 1d and S1). For comparison we also collected native data from crystals grown in 25% w/v PEG 1500 and 0.1 M SPG buffer pH 7.0 (succinic acid, sodium phosphate monobasic monohydrate, glycine). The two resulting structures were virtually identical, showing that the arsenic adduct did not induce structural changes in Sap1. We observe continuous high-quality density for residues 30 to 133 (Fig. 1e).

**Table 1:** Crystallographic Table.

	Native, P2_1_2_1_2_1_	Arsenic
**Data**		
Wavelength (Å)	1.043010	1.043010
Resolution range (Å)	35.61–1.50 (1.55–1.50)	35.39–1.41 (1.46–1.41)
Space group	19, P2_1_2_1_2_1_	19, P2_1_2_1_2_1_
Unit-cell parameters (Å, °)	a = 35.42, b = 40.77, c = 71.23 α = β = γ = 90	a = 35.58, b = 40.83, c = 70.78 α = β = γ = 90
Total reflections	33999 (3326)	40841 (3864)
Unique reflections	17123 (1672)	20477 (1969)
Multiplicity	2.0 (2.0)	2.0 (2.0)
Completeness (%)	99.96 (100.00)	99.49 (96.52)
Mean I/σ (I)	25.64 (6.21)	24.76 (4.62)
Wilson B factor (Å^2^)	12.45	12.26
R_merge_	0.023 (0.123)	0.021 (0.188)
R_means_	0.032 (0.174)	0.030 (0.266)
Mn(I) half-set correlation CC(½)	0.999 (0.961)	0.999 (0.909)
**Refinement**		
Resolution range	35.61–1.5	35.39–1.409
Total number of reflections	17123 (1672)	20476 (1969)
Number of reflections in test set	1314 (128)	1028 (104)
R_work_ (%)	0.163 (0.169)	0.168 (0.229)
R_free_ (%)	0.191 (0.212)	0.198 (0.272)
Real space correlation CC(work)	0.964 (0.948)	0.967 (0.912)
Real space correlation CC(free)	0.946 (0.909)	0.956 (0.847)
No. of atoms		
Non-hydrogen atoms	1022	1005
Macromolecule	882	880
Ligands	0	9
Solvent	140	125
No. of protein residues	105	104
R.m.s.d., bonds (Å)	0.009	0.008
R.m.s.d., angles (°)	0.96	0.97
Ramachandran favored (%)	100.00	100.00
Ramachandran outliers (%)	0.00	0.00
Ramachandran allowed (%)	0.00	0.00
Clash score	0.00	1.71
B factors (Å^2^)		
Average	17.10	17.64
Macromolecule	15.94	16.57
Ligands	0	14.32
Solvent	24.40	25.14

*Statistics for the highest-resolution shell are shown in parentheses.

### Structure of Sap1 reveals similarity to TRF1

**Figure 2:**
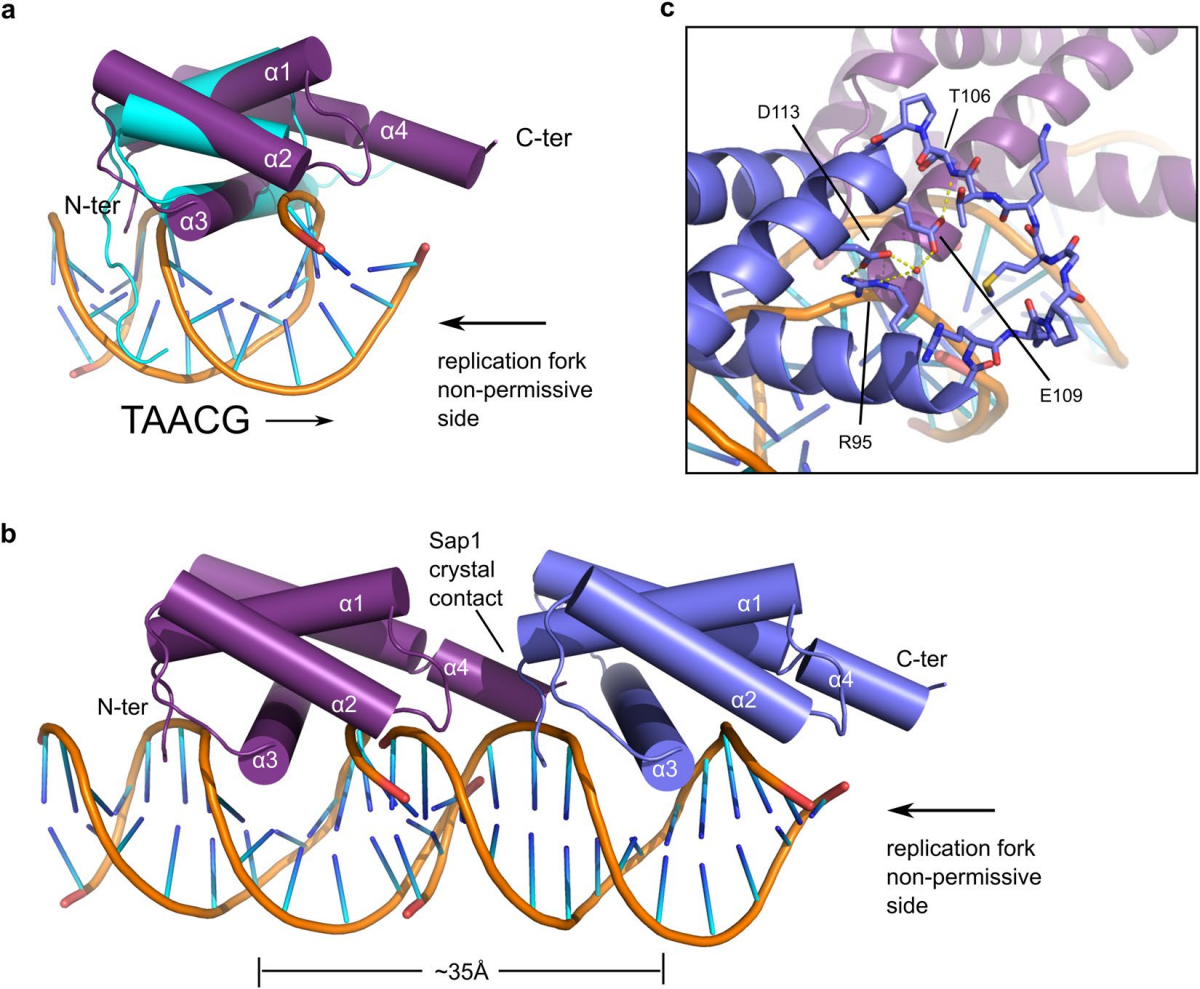
Sap1 crystal contact suggests direct repeat DNA binding mode. (**a**) Superposition of Trf1-DNA complex in cyan (PDBID:2QHB) onto Sap1 DBD. (**b**) Two Sap1 DNA binding domains (purple and blue) are shown as they are arranged in the crystal. The DNA is modeled by superposition of the Trf1-DNA structure. (**b**) Hydrogen bonding network around residue E109 at the crystal contact.

The overall structure of the Sap1 DNA binding domain (DBD) reveals a bundle of four helices, with helices α1-α3 making up a single myb-type helix-turn-helix (HTH) domain (Fig. 1e). In this family of DNA binding domains α3 typically assumes the role of recognition helix and faces into the major groove of the DNA with helices α1 and α2 packed at a right angle on top. The packing between the α1, α2 and α3 helix bundle is imperfect and forms a deep cavity (Fig. S2A). Cys81 is one of the residues lining this cavity and the arsenic adduct previously described occupies the cavity without disturbing the fold of the Sap1 DBD. Sap1 has a fourth, long α4 helix that is linked to helix α3 by the α3-α4 loop, packing on top of α3 against α1. A structural similarity search against the PDB revealed that the telomere binding protein NGTRF1 from Nicotiana tabacum^15^ has a very similar fold and matches all four helices with an RMSD of 2.5 Å (Figs 2a and S2B). Based on the NGTRF1 similarity, α4 corresponds to a C-terminal myb extension domain typical of a distinct class of double-strand telomere DNA binding proteins where it is important for DNA binding^16^. In Sap1, amino acid substitutions in the DBD prevent DNA binding *in vitro*^14^. When we map the mutations found by Arcangioli *et al*.^14^ onto the Sap1 DBD structure, changes at fifteen amino acids essential for Sap1 DNA binding *in vitro* are found in the DNA binding domain, and we see that thereof six are changes to prolines within helices (Fig. S2C,D). Human TRF1 is closely related to *N*. *tabacum* NGTRF1, and we used the cocrystal structure of hTRF1 in complex with DNA for modeling Sap1 binding to DNA (Fig. 2a). This structural model suggests that Sap1 binds with α3 inserted into the major groove of DNA. Our structure as well as the model of Sap1 bound to DNA is entirely consistent with an independent structural analysis of Sap1 that includes an NMR derived model of Sap1 bound to DNA^17^.

The position of the the N-terminal residue 30 in the structure suggests that further N-terminal residues of Sap1 would bind into the DNA minor groove. Because truncation of N-terminal 22 residues allows nuclease access to the 5′ end of the TAACG Sap1 binding motif^11^ we propose that the TAACG motif runs from the N-terminus of Sap1 towards the C-terminus at the end of the α4 helix (Fig. 2a). On Ter1, where Sap1 stalls the replication machinery, this model predicts that the α4 helix points towards the oncoming replisome (Fig. 2a).

### Sap1 crystal packing suggests a model for head-to-tail oligomerization of Sap1 on DNA

When we analyzed the packing of Sap1 molecules inside the crystal we observed an intriguing Sap1 dimerization interface (Fig. 2b). The translational symmetry operation creating this interface joins N- and C-termini of the Sap1 DBDs in a head-to-tail fashion and positions the recognition helices about 35 Å apart. This corresponds to the helical repeat length of DNA, and we therefore propose that this dimerization mode is potentially involved in binding of Sap1 dimers to consecutive major grooves in a direct repeat mode (Fig. 2b). This mode of DNA binding has been proposed for Sap1’s binding to the replication fork stalling site Ter1 based on footprinting and DNA protection assays^11^. The interface we observe consists on one side of residues belonging to the α1-α2 loop and to the C-terminus of the α4 helix. They pack against the N-terminus of the α1 helix and a cradle formed by residues of α3, the α3-α4 loop and the N-terminus of α4 of a neighboring molecule (Fig. 2c).

The DNA binding activity of Sap1 depends strongly on the well-characterized coiled-coil dimerization domain that is found C-terminal to the Sap1 DBD, and the DBD alone does not bind to the Ter1 and synthetic direct repeat sites^11,18^. When we extend the DNA based on the Trf1 structural homology to model the direct repeat binding mode of the Sap1 dimer, we observe that the end of the C-terminus of the α4 helix clashes with the phosphate backbone of the minor groove that is bridged by the dimerization interface. It is conceivable that the coiled-coil, which attaches to the C-terminus of α4, induces structural changes locally that facilitate DNA binding.

### Oligomerization interface of Sap1 harbors *abp1Δ cbh1Δ* suppressor mutations

**Figure 3:**
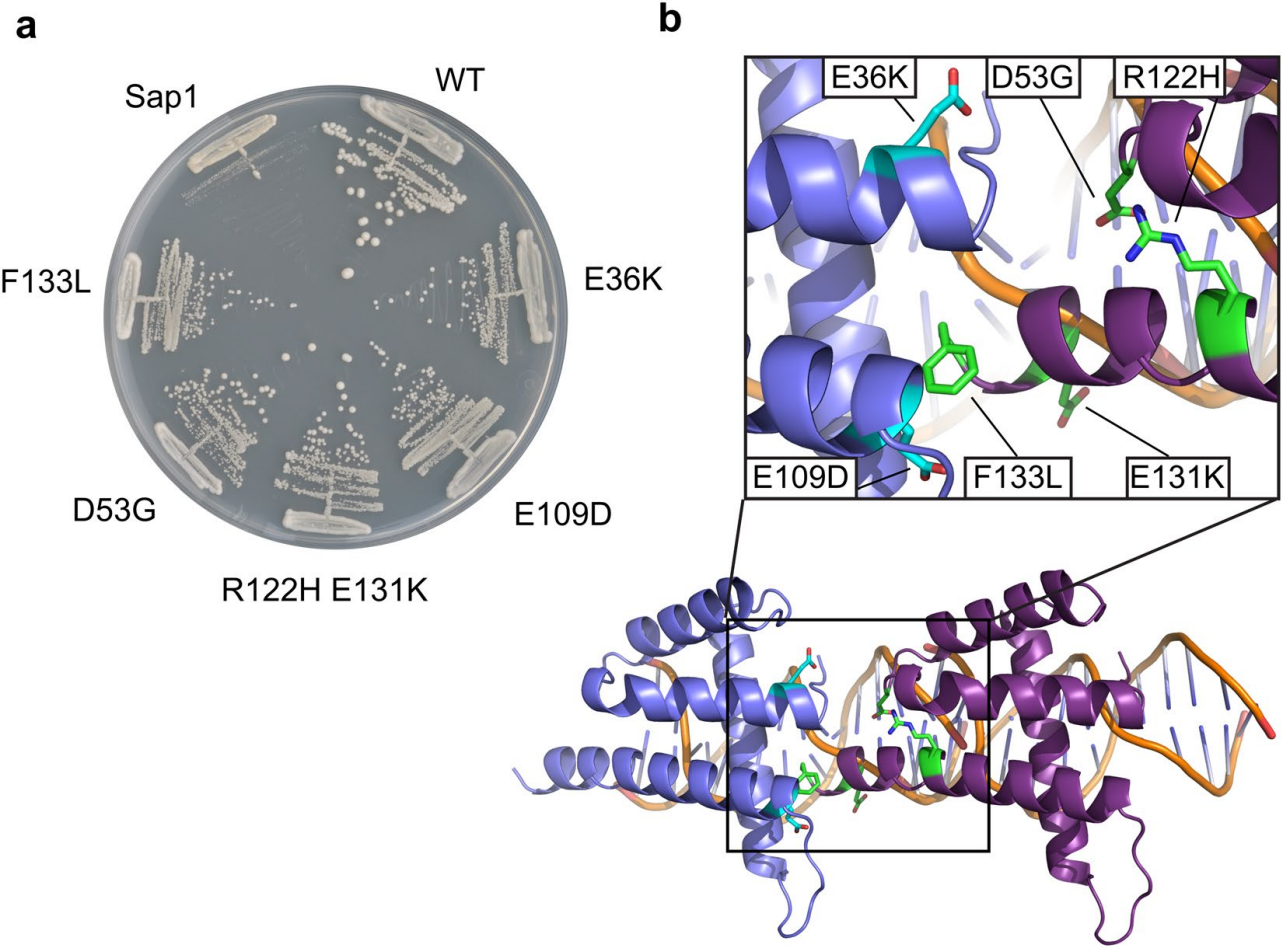
Mutations affecting the Sap1 crystal dimerization interface suppress the requirement for CENP-B proteins. (**a**) Comparison of growth of abp1Δ/cbh1Δ double mutants expressing the wild-type sap1+ gene (‘Sap1’) or suppressor alleles producing Sap1 proteins with the indicated amino acid substitutions, on YES medium. A wild-type abp1+ cbh1+ sap1+ strain is shown for comparison (‘WT’). (**b**) Mapping of mutations in B onto the Sap1 crystal contact. The mutant amino acids are colored in green for one monomer or cyan for the other monomer in the Sap1 dimer displayed below.

Since our observation of the dimerization interface is limited to the DBD in the crystallization lattice, we desired to investigate the functional significance of this interaction. Intriguingly, the Sap1-c mutation that suppresses cell death in CENP-B deletion mutants^13^ maps to residue E109 at the N-terminus of α4. The growth defect in the CENP-B mutants is caused by stalled replication forks at transposons, and the Sap1-c E109D mutation suppresses stalling by weakening the Sap1 mediated replication fork blocks. The analysis of the Sap1 DBD structure shows that E109 is part of an intricate hydrogen-bond network (Fig. 2c) that forms the basis of the Sap1 direct repeat dimerization and crystal packing interface formed by α3, the α3-α4 loop and the N-terminus of α4. Indeed, we failed to obtain crystals for a Sap1 E109D mutant while the wild type Sap1 DBD readily produced high quality crystals under many different conditions. This finding suggests that the interface we observe contributes to the Sap1 replication fork barrier. In order to further test this hypothesis, we searched for additional suppressor mutants of the CENP-B phenotype. Screens were conducted with strains lacking both CENP-B homologs Cbh1 and Abp1, similar to the screen that produced the Sap1-c mutant^13^. Four additional suppressors were obtained in *sap1*, all of which give rise to amino acid changes at the predicted oligomerization interface: E36K; D53G; R122H E131K; and F133L, the latter obtained twice independently in unmutagenized populations of ZB724 and TV418 (Figs 3 and S3A for retesting). Importantly, these amino acid substitutions are distributed between the two surfaces buried upon Sap1 dimerization, supporting the notion that these mutations destabilize the Sap1 head-to-tail interaction and thereby alleviate the need for CENP-B proteins.

We tested DNA binding affinity for isolated Sap1 and Sap1-c proteins (Fig. S3B,C) in order to determine whether DNA binding affinity is affected in the Sap1c mutant as suggested by experiments in whole cell lysates^13^. In contrast to these findings we do not observe any loss of affinity for DNA and instead observe a two-fold increase in affinity for the Sap1-c mutant.

### Mutations in the Sap1 direct repeat interface are lethal

To further probe the requirements for dimerization or oligomerization at the predicted interface *in vivo*, mutations were introduced to perturb the hydrogen-bonding network (E109K; K132E) or hydrophobic interactions (L34A L112A and F133A Y129A). These mutations were introduced in a loop-in loop-out strategy^19^. For each mutation, a mutated *sap1* gene was inserted at the *sap1* locus in tandem with wild-type *sap1*+. An intervening *ura4*+ gene was integrated at the same time and used at first to select for integration (loop-in, selecting for uracil prototrophy) and subsequently to select for excision (loop-out, selecting for FOA resistance). In this strategy, loop-outs produce either a mutated *sap1* allele or restore the wild-type gene, each event occurring with a probability determined by the length of flanking homology on each side of the mutation. Here, the homology length was biased so as to favor recovery of the mutants over wild-type, yet only wild-type were recovered following loop-out, the E109K, K132E, L34A L112A and F133A Y129A mutants were not recovered showing the essentiality of these amino acids. The same recombination regime effectively replaced codons in similar locations, for example K132A was viable, and suppressor mutations could also be reintroduced for retesting. We conclude that particular amino acid changes are not tolerated at the head-to-tail interface, indicating its importance for the essential function of Sap1.

## Discussion

Here we determined and analyzed the crystal structure of Sap1. It shows high similarity to Myb proteins such as Trf1 that play important roles in telomere maintenance. While there is no evidence to support a role for Sap1 in telomere biology, the two proteins might share some functions in the control of DNA replication^20^.

Our structure is entirely consistent with a previously solved structure of Sap1^17^ where it was interpreted in the context of Sap1 function at replication origins. Our work analyzes the structure from a very different angle, as we focus on the head-to-tail arrangement of Sap1 DBDs in the crystal lattice, which is compatible with binding to a tandem repeat of cognate DNA binding sites as found at Ter1 sites in rDNA repeats where Sap1 establishes a replication fork block site^9^. Intriguingly, the head-to-tail dimerization interface also harbors a mutation that alleviates the growth defects caused by loss of CENP-B proteins^13^, and our screening for suppressors found more mutations mapping to the dimerization interface.

It remains to be established, how Sap1 interface mutants alleviate the CENP-B growth defects. DNA binding activity of Sap1-c to Ter1 is not affected when compared to wild type Sap1. Furthermore, Sap1-c is also functional in replication for blocking activity at Ter1. This suggests that these mutations in dimerization interface residues produce subtle changes in the replication fork block, which only have an effect in the context of the Tf2 transposons blocks. We note that there seems to be a fine line for mutations that suppress the CENP-B phenotype before they become toxic, since mutations to different amino acids in the same positions (E109K, K132E, L34A L112A and F133A Y129A) within the dimerization interface are lethal.

The structure was solved using a serendipitous arsenic adduct (dimethylarsenate) that bound to residue Cys81. This adduct did not alter the structure and shows that there is a cavity inside the Sap1 protein that can accommodate an organic molecule. The cavity’s connection to the solvent is too narrow in our structure to let pass a water molecule. However, the fact that cacodylic acid, which reacts normally with solvent-accessible cysteines, is found inside the cavity indicates that the pocket may be accessible from the solvent at least temporarily. Similar adducts have been observed previously in protein structures^21,22^. The *E. coli* Tus-Ter system has been proposed to rely on a “mouse-trap” mechanism where a cytosine base is trapped in a pocket of the Tus protein to stall the replication fork. We speculate that the cavity in Sap1 might play a role in a similar mechanism in *S*. *pombe*.

Our functional analysis highlights the critically important role of two surface patches on Sap1 that are not directly involved in DNA binding but that pack against each other in the crystal environment. The mutants and the structural analysis will be helpful in revealing the intricate mechanisms that act at the replication fork.

## Materials and Methods

### General fission yeast handling and molecular biology techniques

Media, genetic manipulations of *S*. *pombe* and genomic DNA preparations were as described^23^. Strains and their genotypes are reported in Table S1. Oligonucleotides and Geneblocks (IDT) are reported in Table S2. DNA amplification for cloning was carried out with Phusion (Thermo Fisher Scientific); 12 cycles were used to amplify plasmid DNA with ~2 ng template per reaction and 18 cycles to amplify *S*. *pombe* genomic DNA with ~200 ng template per reaction.

### Plasmid constructions for Sap1 expression in *E*. *coli*

The pET28a-SUMO-His_6_ vector^24^ was amplified by inverse PCR with GTO-569 and GTO-570 and portions of the *sap1*^+^ ORF were amplified in parallel from *S*. *pombe* genomic DNA with ends that overlapped pET28a-SUMO-His_6_ and allowed Gibson assembly^25^. Sap1 (25–150) was amplified with GTO-563 and GTO-565 (amplicon size 375 bp), and Sap1 (18–133) was amplified with oligonucleotides O.10A and O.109. The pET28a-SUMO-His_6_ expression system takes advantage of the SUMO ULP1 deconjugating enzyme to very specifically cleave off the SUMO-His_6_ tag from purified SUMO-His_6_ fusion proteins.

### Protein expression and purification

pET28a-SUMO-His_6_ clones encoding portions of Sap1 were expressed in *E*. *coli* strain C2566 (*fhuA*2 *lacZ::T7 gene1* [*lon*] *ompT gal sulA11 R*(*mcr*-*73::miniTn10–TetS*)*2 [dcm] R*(*zgb*-210*::Tn10–TetS*) *endA1 Δ*(*mcrC*-*mrr*)*114::IS10*, NEB) at 37 °C to produce SUMO-His_6_-Sap1 fusions. Protein expression was carried out in 2 l LB medium containing 50 μg/ml kanamycin; it was induced for 2 h when the cells had grown to an OD600 of 0.6–0.7 by the addition of isopropyl-β-d-1-thiogalactopyranoside (IPTG) to a final concentration of 0.2 mM. Cells were harvested and resuspended in 20 ml (50 mM sodium phosphate pH 7.4, 300 mM NaCl, 0.1% Triton X-100) before being lysed by sonication followed by ultracentrifugation at 20,000 g for 30 min, 4 °C. The cleared lysates were filtered by Syringe Minisart filters pore size 0.2 μm (Sigma-Aldrich) and the His_6_-tagged proteins were purified on TALON® Metal Affinity Resin columns (Clontech, 2 ml bed volume) equilibrated in 50 mM sodium phosphate pH 7.4, 300 mM NaCl. Proteins were bound to the column at 4 °C with constant mixing for 30 min followed by a wash with 2x bed volumes of 50 mM sodium phosphate pH 7.4, 300 mM NaCl, 20 mM Imidazole before elution in 1 ml fractions with 50 mM sodium phosphate pH 7.4, 300 mM NaCl, 150 mM Imidazole. Eluted fractions with protein concentration above 200 nM were pooled and dialysed O/N against 10 mM N-2-hydroxyethylpiperazine-N9-2-ethanesulfonic acid, pH 7.4 (HEPES) at 4 °C. In the same step, the Sap1 polypeptides were deconjugated from the SUMO-His_6_ tag with the ULP1-His_6_ protease^24^. Samples were concentrated using ultra centrifugal filter units Ultra-15, MWCO 30 kDa (Amicon) prior to size-exclusion chromatography on a Superdex 75 10/300 G column (GE Healthcare). The Superdex column was connected to a TALON resin column for reverse purification in order to remove His_6_-SUMO and ULP1-His_6_. Purity was confirmed by SDS-PAGE and concentration was determined by measurements at A280 nm with a NanoDropTM 1000 spectrophotometer (Thermo Fisher Scientific). Between 20–30 mg was obtained for each polypeptide.

### Limited Proteolysis

Sap1 protein construct 1–212 at a concentration of 10 mg/ml in buffer containing 10 mM HEPES pH, 150 mM NaCl and 1 mM BME was incubated with different amounts of thermolysin from 10 ug to 0.1 ng at room temperature. After 30 min of thermolysin treatment, buffer containing EDTA was added to stop the reaction and mixed with loading dye. After boiling the samples for 5 min, limited trypsin digestion was verified by 12% SDS-PAGE and Coomassie brilliant blue staining.

### Mass spectrometry analysis

The stable fragment of Sap1 obtained following thermolysin treatment was purified by reverse phase HPLC on a ZORBAX 300SB C18 column (Agilent). The molecular mass of the Sap1 stable fragment was determined by MALDI-TOF mass spectrometry. For MALDI-TOF the sample was spotted using sinapinic acid [10 mg/ml] in [70:30] acetonitrile-trifluoroacetic acid [0.1%]) on a MTP 384 target plate (Bruker Daltonics). An Autoflex III MALDI-TOF/TOF spectrometer (Bruker Daltonics) was used in linear mode with the following settings: 5,000- to 50,000-Da window; linear positive mode; ion source 1, 20 kV; ion source 2, 18.5 kV; lens, 9 kV; pulsed ion extraction of 120 ns; and high gating ion suppression up to 1,000 *M*_r_. Mass calibration was performed externally with Bruker’s Protein 1 standard calibration mixture (Bruker Daltonics). Data acquisition was performed using the FlexControl 3.0 software program (Bruker Daltonics), and peak searching and subsequent spectral analysis were performed using FlexAnalysis 3.0 software (Bruker Daltonics).

### Crystallisation and structure processing

The following two portions of Sap1 were cloned and expressed as described above: aa 25–150 (fragment lacking the secondary dimerization domain, containing the smallest portion of the primary dimerization domain needed for DNA binding reported so far (Ghazvini *et al*. 1995) and aa 18–133. High resolution diffracting crystals of Sap1 (aa 18–133) were obtained by sitting drop vapour diffusion in 0.1 M PCTP buffer (sodium propionate, sodium cacodylate trihydrate, bis-tis propane) pH 7.0, 25% PEG 1500 or in 0.1 M SPG buffer (succinic acid, phosphate, glycine) pH 7.0, 25% PEG 1500. Prior to transport, the crystals were harvested and flash-frozen in liquid nitrogen. Data were collected at the Swiss Synchrotron Light Source (SLS) PX-III beamline.

### Electrophoretic mobility shift assay (EMSA)

Full length Sap1 and Sap1-c (E109D) in dilution buffer containing 10 mM HEPES pH 7.5, 100 mM NaCl and 5% glycerol were serially diluted by 2 fold from 5120 nM to 0.68 nM concentration. Diluted samples were mixed with ^32^P labelled Ter1 or Tf2 oligonucleotides in Binding Buffer (20 mM HEPES pH 7.6, 50 mM potassium chloride, 3 mM magnesium chloride, 8% glycerol and 0.2% NP-40). Samples were incubated for 30 min on ice, loaded onto a pre-run 10% polyacrylamide gel and run for 3 h at 200 V in 0.25xTBE. The gels were dried at 60 °C for 5–6 hours (Biorad gel dryer) and exposed overnight for phosphorimaging (Biorad).

### Directed mutagenesis of *sap1*

Mutations of interest were introduced into the *S*. *pombe* chromosome at the endogenous *sap1*^+^ locus using a loop-in/loop-out strategy^19^ with plasmids that contained the *S*. *pombe ura4*^+^ gene and a mutagenized s*ap1* ORF. A backbone plasmid was first produced by amplifying the *sap1*^+^ ORF and its flanking sequences from genomic DNA of wild-type strain 968 with oligonucleotides GTO-815 and GTO-816 and by cloning the amplicon into the EcoRV site of plasmid pJET1.2 (Thermo Fisher Scientific). Construction of the backbone plasmid was completed by inserting the *S*. *pombe ura4*^+^ gene into the XmaI-NarI sites at one edge of the *sap1*^+^ insert, creating plasmid pMJ13. The source of *ura4*^+^ for this cloning was pGT189, a pUC18^26^ derivative bearing the 1.8 kb genomic *Hin*dIII fragment that contains *ura4*^+^, released here with XmaI and ClaI. pMJ13 was mutagenized with Geneblock gGT4 to introduce the *sap1*-*L34A*-*L112A* mutations that are far apart from each other, or with mutagenic oligonucleotides for more localized mutagenesis. In order to obtain enough material, gGT4 was first cloned into pJET1.2. The mutations were then transferred to pMJ13 by replacing the small BamHI-HpaI fragment of pMJ13 with the corresponding pJET1.2-gGT4 fragment. The *sap1*-*K132A*, *sap1*-*E109K*, *sap1*-*K132E*, *sap1*-*Y129A*-*F133A* and *sap1*-*C81M/R* mutations were introduced into pMJ13 by inverse PCR with mutagenic oligonucleotides, respectively GTO-844 and GTO-845; GTO-848 and GTO-849; GTO-850 and GTO-851; GTO-854 and GTO-853; and GTO-852 and GTO-853. The *sap1*-*D53G* and *sap1*-*R122H*-*E131K* suppressor alleles were amplified by PCR with strain ZB786 and ZB787 and oligonucleotides GTO-815 and GTO-816: the mutations were introduced into pMJ13 by replacing the BamHI-BstEII fragment. Plasmids were linearized with BstEII for insertion at the wild-type *sap1*^+^ locus in strain PG3764 in the case of mutations designed to test the crystal structure or linearized with HpaI for insertion at *sap1*^+^-*5FLAG*-*hph1* in strain MJ75, to retest suppressor mutations. The *sap1*^+^-*5FLAG*-*hph1* allele in strain MJ75 was obtained by swapping *kanR* for *hph1* in the *sap1*^+^-*5FLAG*-*kanR* allele from^27^. BstEII and HpaI cut only once in the mutagenized plasmids, at the very end of the *sap1* ORF in the case of BstEII and internal to the ORF in the case of HpaI, leaving in both cases homology on both sides of the cut for chromosomal insertion. Loop-in strains containing tandem wild-type and mutant copies of the *sap1* gene at the endogenous *sap1* locus were obtained by homologous integrations of the digested plasmids in the recipient *ura4*-*D18* strains PG3764 or MJ75. Stable Ura^+^ transformants with correct integrations were identified by PCR with GTO-906 and GTO-907 for PG3764, and with GTO-906 and GTO-1362 or GTO-1376 and GTO-1362 for MJ75. Presence of a mutated looped-in allele was verified by sequencing PCR products with GTO-1377. Loop-out strains in which either the wild-type or mutated *sap1* allele remained as sole genomic copy were subsequently selected on 5′-fluoroorotic acid (FOA)-containing medium and characterized by PCR with oligonucleotides GTO-816 and GTO-855 before being sequenced with GTO-855. In this loop-in/loop-out strategy, according to the length of 5′ and 3′ flanking homology regions on either side of the introduced mutation(s), recovery of only wild-type loop-out alleles can reveal lethal mutations. Here, mutant loop-out strains were recovered for *sap1*-*K132A*, *sap1*-*D53G*, and *sap1*-*R122H*-*E131K*, but not for *sap1*-*E109K*, *sap1*-*K132E*, *sap1*-*C81M/R*, *sap1*-*L34A*-*L112A*, or *sap1*-*F133A*-*Y129A*.

### Suppressor screens

Two screens were performed to identify Sap1 suppressors of the *abp1*Δ *cbh1*Δ slow growth phenotype in addition to the previously identified *sap1*-*c* mutation. In a first approach, strain ZB724 was streaked for single colonies on YEA plates and grown for one week at 32 °C. In order to ensure the absence of suppressors in the initiating culture, small colonies were picked, and their doubling time in liquid YEA was measured and compared to TV418. Colonies of ZB724 showing the same doubling time as TV418 (~11–14 h) were selected, grown to OD_600_ ~ 0.5 and mutagenized with nitrosoguanidine in conditions resulting in ~50% survival. Briefly, cells were washed in TM6 buffer (50 mM Tris Maleate pH 6), resuspended at 2.5 × 10^8^ cells/ml in TM6 buffer with 428 µg/ml nitrosoguanidine, incubated for 13 minutes, pelleted and washed in TM6 buffer. After two additional washes in liquid YEA medium, cells were plated on YEA at a density of 10^3^ cells/plate and grown at 32 °C for 1 week. Large colonies were picked and crossed with *abp1*^+^ *cbh1*^+^ cells, by mass sporulation onto YEA plates containing 200 µg/l G418 and 200 µg/l nourseothricin to select for *abp1*Δ *cbh1*Δ progeny. Fast-growing mutants showing 1:1 segregation for colony size were crossed again with *abp1*^+^ *cbh1*^+^ cells, and the *sap1* gene was amplified and sequenced from both fast and slow growing *abp1*Δ *cbh1*Δ progeny to identify mutants for which fast growth correlated with the presence of a mutation in *sap1*. The mutant strains ZB762 (*sap1*-*E36K*), ZB786 (*sap1*-*D53G*), and ZB787 (*sap1*-*R122H*-*E131K*) were obtained in this screen.

In a second approach, 20 single colonies of each ZB724 and TV418 were isolated and patched on YES. Each patch was used to inoculate an 8 ml YES culture that was incubated with vigorous shaking at 33 °C. The following weeks, cultures were diluted every third or fourth day and in the same interval plated on YES with a cell density of 10^6^ cells per plate until large, fast-growing colonies appeared. A minimum of two large colonies were chosen for each culture and re-streaked for isolated colonies. Isolated colonies were patched and propagated in 8 ml YEL cultures for DNA preparation. The *sap1* ORF was amplified with GTO-815 and GTO-855 and sequenced (Macrogen). In this approach, only one mutation was found in the *sap1* gene (F133L), but this mutation arose independently in both strains (MJ42 and MJ62).

Suppression by *sap1*-*D53G* and *sap1*-*R122H*-*E131K* was further tested by introducing the mutations in MJ75 by loop-in loop-out of mutagenized plasmids, creating strain MJ77 (*sap1*-*D53G*-*5FLAG*-*hph1*) and strain MJ89 (*sap1*-*R122H*-*E131K*-*5FLAG*-*hph1*). Diploids with ZB724 were created using intra-allelic complementation between *ade6*-*210* and *ade6*-216. Following tetrad dissection on YES and colony formation at 33 °C, the plates were photographed and replica-plated onto selective media to assign genotypes and to determine whether the reconstructed mutant alleles suppressed the poor growth of *abp1*Δ *cbh1*Δ double mutants. A cross between MJ75 (*sap1*-*5FLAG*-*hph1*) and ZB724 was processed in parallel to verify that suppression was not caused by the 5FLAG tag. YES plates are shown in Fig. S3.

### Accession codes

Coordinates and structure factors have been deposited in the Protein Data Bank under accession codes 6EXU (arsenic) and 6EXT (native).

## Electronic supplementary material

Supplementary Information

## References

[1] Labib K, Hodgson B (2007). Replication fork barriers: pausing for a break or stalling for time?. EMBO Rep..

[2] Leman AR, Noguchi E (2013). The replication fork: understanding the eukaryotic replication machinery and the challenges to genome duplication. Genes.

[3] Berghuis BA (2018). What is all this fuss about Tus? Comparison of recent findings from biophysical and biochemical experiments. Crit. Rev. Biochem. Mol. Biol..

[4] Neylon C, Kralicek AV, Hill TM, Dixon NE (2005). Replication termination in Escherichia coli: structure and antihelicase activity of the Tus-Ter complex. Microbiol. Mol. Biol. Rev..

[5] Mulcair MD (2006). A molecular mousetrap determines polarity of termination of DNA replication in *E. coli*. Cell.

[6] Voineagu I, Narayanan V, Lobachev KS, Mirkin SM (2008). Replication stalling at unstable inverted repeats: interplay between DNA hairpins and fork stabilizing proteins. Proc. Natl. Acad. Sci. USA.

[7] Bada M, Walther D, Arcangioli B, Doniach S, Delarue M (2000). Solution structural studies and low-resolution model of the Schizosaccharomyces pombe sap1 protein. J. Mol. Biol..

[8] Arcangioli B, Klar AJ (1991). A novel switch-activating site (SAS1) and its cognate binding factor (SAP1) required for efficient mat1 switching in Schizosaccharomyces pombe. EMBO J..

[9] Krings G, Bastia D (2005). Sap1p binds to Ter1 at the ribosomal DNA of Schizosaccharomyces pombe and causes polar replication fork arrest. J. Biol. Chem..

[10] Mejía-Ramírez E, Sánchez-Gorostiaga A, Krimer DB, Schvartzman JB, Hernández P (2005). The mating type switch-activating protein Sap1 Is required for replication fork arrest at the rRNA genes of fission yeast. Mol. Cell. Biol..

[11] Krings G, Bastia D (2006). Molecular architecture of a eukaryotic DNA replication terminus-terminator protein complex. Mol. Cell. Biol..

[12] Jacobs JZ (2015). Arrested replication forks guide retrotransposon integration. Science.

[13] Zaratiegui M (2011). CENP-B preserves genome integrity at replication forks paused by retrotransposon LTR. Nature.

[14] Arcangioli B, Ghazvini M, Ribes V (1994). Identification of the DNA-binding domains of the switch-activating-protein Sap1 from S.pombe by random point mutations screening in E.coli. Nucleic Acids Res..

[15] Ko S (2008). Structure of the DNA-binding domain of NgTRF1 reveals unique features of plant telomere-binding proteins. Nucleic Acids Res..

[16] Karamysheva ZN, Surovtseva YV, Vespa L, Shakirov EV, Shippen DE (2004). A C-terminal Myb extension domain defines a novel family of double-strand telomeric DNA-binding proteins in Arabidopsis. J. Biol. Chem..

[17] Guan L (2017). Sap1 is a replication-initiation factor essential for the assembly of pre-replicative complex in the fission yeast Schizosaccharomyces pombe. J. Biol. Chem..

[18] Ghazvini M, Ribes V, Arcangioli B (1995). The essential DNA-binding protein sap1 of Schizosaccharomyces pombe contains two independent oligomerization interfaces that dictate the relative orientation of the DNA-binding domain. Mol. Cell. Biol..

[19] Gao J (2014). Rapid, efficient and precise allele replacement in the fission yeast Schizosaccharomyces pombe. Curr. Genet..

[20] Higa, M., Fujita, M. & Yoshida, K. DNA Replication Origins and Fork Progression at Mammalian Telomeres. *Genes* **8** (2017). 10.3390/genes8040112 PMC5406859 28350373

[21] Brooks MA, Ravelli RBG, McCarthy AA, Strub K, Cusack S (2009). Structure of SRP14 from the Schizosaccharomyces pombe signal recognition particle. Acta Crystallogr. D Biol. Crystallogr..

[22] Liu X, Zhang H, Wang X-J, Li L-F, Su X-D (2011). Get phases from arsenic anomalous scattering: de novo SAD phasing of two protein structures crystallized in cacodylate buffer. Plos One.

[23] Ekwall, K. & Thon, G. Genetic Analysis of Schizosaccharomyces pombe. *Cold Spring Harb*. *Protoc*. **2017**, db.top079772 (2017). 10.1101/pdb.top079772 28765303

[24] Mossessova E, Lima CD (2000). Ulp1-SUMO crystal structure and genetic analysis reveal conserved interactions and a regulatory element essential for cell growth in yeast. Mol. Cell.

[25] Gibson DG (2009). Enzymatic assembly of DNA molecules up to several hundred kilobases. Nat. Methods.

[26] Norrander J, Kempe T, Messing J (1983). Construction of improved M13 vectors using oligodeoxynucleotide-directed mutagenesis. Gene.

[27] Noguchi C, Noguchi E (2007). Sap1 promotes the association of the replication fork protection complex with chromatin and is involved in the replication checkpoint in Schizosaccharomyces pombe. Genetics.

