# Supplementary material for: Structure of the replication regulator Sap1 reveals functionally important interfaces
Source: Sci Rep. 2018 Jul 19;8:10930. doi: 10.1038/s41598-018-29198-9 (PMC6053445; doi:10.1038/s41598-018-29198-9)
Supplement: Supplementary file 1 — Supplementary Information [file 41598_2018_29198_MOESM1_ESM.pdf]

Supplemental Information

Structure of the replication regulator Sap1 reveals functionally important interfaces

Maria M. Jørgensen[1%], Babatunde Ekundayo[2%], Mikel Zaratiegui[3], Karen Skriver[1], Geneviève Thon[1*], Thomas Schalch[2,4*]

[1] Department of Biology, University of Copenhagen, Copenhagen, Denmark

[2] Department of Molecular Biology, Science III, Institute of Genetics and Genomics of Geneva (iGE3), University of Geneva, CH-1211 Geneva 4, Switzerland

[3] Department of Molecular Biology and Biochemistry, Rutgers University, Piscataway, USA

[4] Leicester Institute for Structural and Chemical Biology, Department of Molecular and Cell Biology, University of Leicester, Leicester, LE1 9HN, UK

[%] Authors contributed equally

[*]Corresponding authors:

Email: gen@bio.ku.dk, Tel: +45 35 33 01 98

Email: thomas.schalch@leicester.ac.uk, Tel: +44 116 229 7120

Supplemental Figures S1-S3
Supplemental Tables S1-2

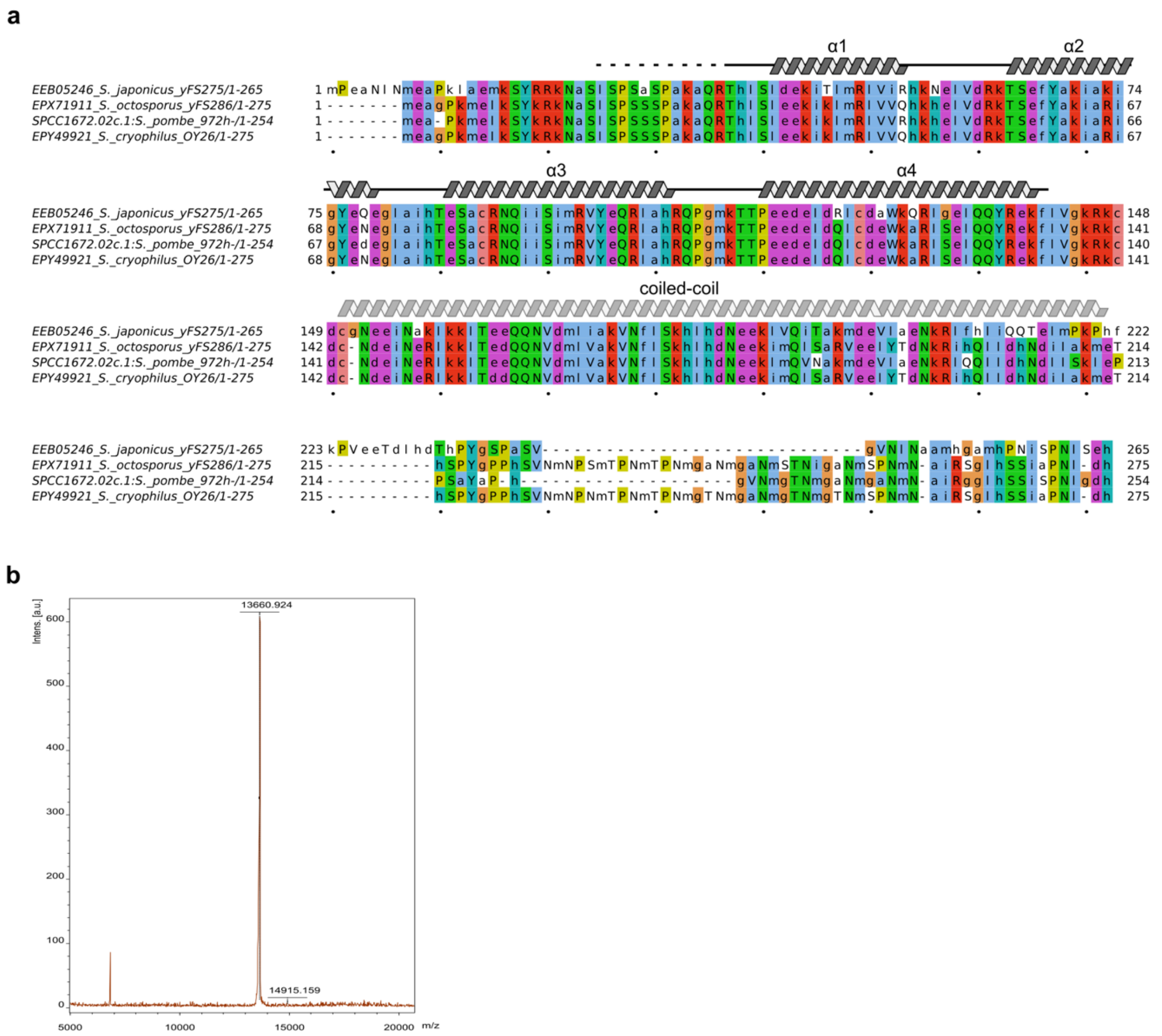

Figure S1: (A) Sequence alignment of *Schizosaccharomyces* Sap1 proteins. The secondary structure annotation is derived from the Sap1 crystal structure. (B) MALDI peak of the thermolysin limited proteolysis fragment in Fig. 1C.

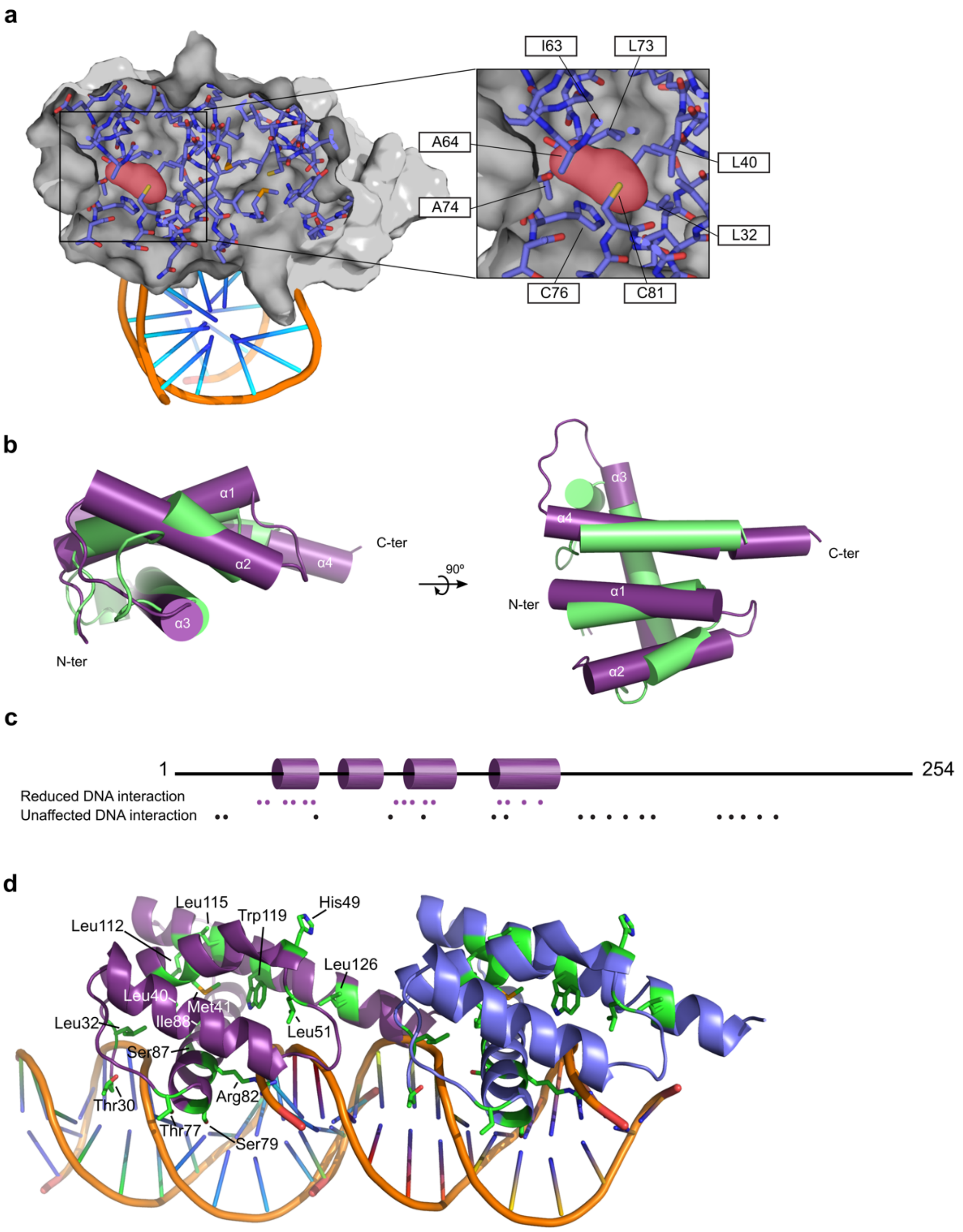

Figure S2: The Sap1 DNA binding domain resembles telomere-binding proteins. (A) Structure of the native Sap1 DNA binding domain with putative position of DNA modeled based on structural homology to Trf1 as in Fig. 2. The deep pocket is colored in red. This pocket is filled by the dimethylarsinoyl adduct to C81 in the derivative crystal. (B) Superposition of Sap1 DBD onto NGTRF1 (PDBID:2CKX). (C) Mapping of amino acids essential to Sap1-DNA interaction in vitro onto the crystal structure. Amino acid substitutions leading to reduced (purple dots) or unaffected (black dots) DNA interaction[1] are shown on a linear representation of Sap1. (D) The amino acids whose substitution leads to loss of DNA binding[1] are shown as green sticks in a cartoon representation of the Sap1 structure.

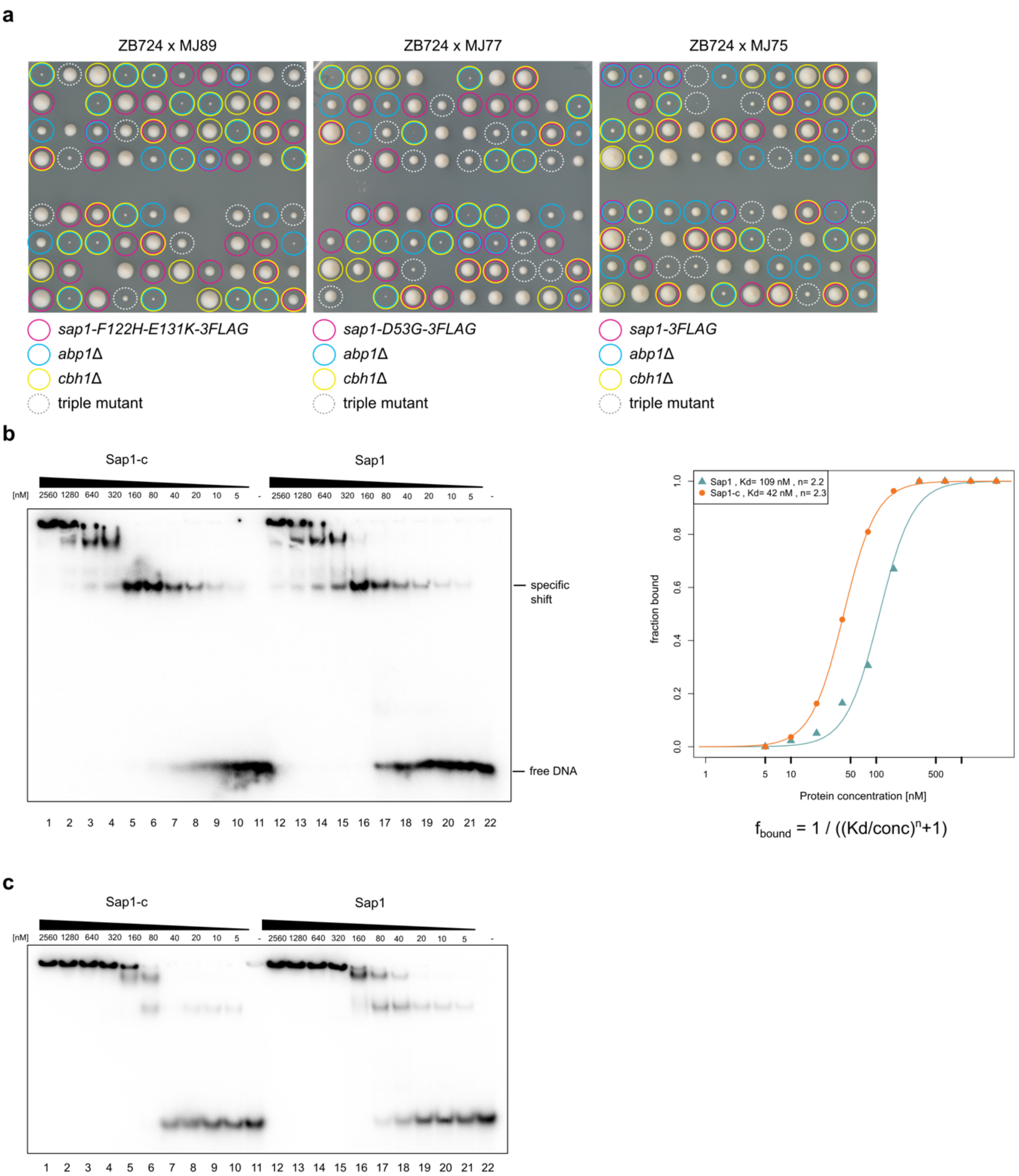

Figure S3: Analysis of Sap1 suppressors. (A) Tetrad dissection of diploids of the indicated strains retesting suppression of the abp1D cbh1D phenotype by mutations in sap1. (B) Electrophoretic mobility shift assay with radiolabeled Ter1 fragment for Sap1 and Sap1-c with corresponding fit of binding isotherm to gel data. (C) EMSA as (D) for radiolabeled Tf2 DNA fragment.

Table S1. Strain genotypes

Strain	Genotype
MJ15	*h^{90} sap^{+}[sap1-C81M loop-in] ura4-D18 ade6-DN/N*
MJ17	*h^{90} sap^{+}[sap1-K132A loop-in] ura4-D18 ade6-DN/N*
MJ18	*h^{90} sap^{+}[sap1-F133A Y129 loop-in] ura4-D18 ade6-DN/N*
MJ26	*h^{90} sap1-K132A ura4-D18 ade6-DN/N*
MJ28	*h^{90} sap^{+}[sap1-L34A L112A loop-in] ura4-D18 ade6-DN/N*
MJ30	*h^{90} sap^{+}[sap1-C81R loop-in] ura4-D18 ade6-DN/N*
MJ31	*h^{90} sap^{+}[sap1-K26A-R29A loop-in] ura4-D18 ade6-DN/N*
MJ42	*h^{+} sap1-F133L abp1Δ::KanMX6 cbh1Δ::Nat his3Δ leu1-32 ura4-DS/E ade6-210*
MJ62	*h^{+} sap1-F133L abp1Δ::LEU2 cbh1Δ::LEU2 leu1-32 ura4-DS/E ade6-216*
MJ77	*h^{90} sap1-D53G-5FLAG-hph1 leu1-32 ura4-D18 ade6-216*
MJ75	*h^{90} sap1-5FLAG-hph1 leu1-32 ura4-D18 ade6-216*
MJ88	*h^{90} sap1-E36K-5FLAG-hph1 leu1-32 ura4-D18 ade6-216*
MJ89	*h^{90} sap1-R122H-E131K-5FLAG-hph1 leu1-32 ura4-D18 ade6-216*
PG3764	*h^{90} ura4-D18 ade6-DN/N*
TV418	*h^{+} abp1Δ::LEU2 cbh1Δ::LEU2 leu1-32 ura4-DS/E ade6-216*
ZB371	*h^{+} sap1-E109D abp1Δ::LEU2 cbh1Δ::LEU2 leu1-32 ura4-DS/E ade6-216*
ZB724	*h^{+} abp1Δ::KanMX6 cbh1Δ::Nat his3Δ leu1-32 ura4-DS/E ade6-210*
ZB762	*h^{+} sap1-E36K abp1Δ::KanMX6 cbh1Δ::Nat his3Δ leu1-32 ura4-DS/E ade6-216*
ZB786	*h^{+} sap1-D53G abp1Δ::KanMX6 cbh1Δ::Nat his3Δ leu1-32 ura4-DS/E ade6-216*
ZB787	*h^{+}sap1-R122H-E131K abp1Δ::KanMX6 cbh1Δ::Nat his3Δ leu1-32 ura4-DS/E ade6-216*

Table S2. Oligonucleotides

Identifier	Sequence
GTO-563	ACAGAGAACAGATTGGTGGAGCCAAGGCACAGCGTACTCAC
GTO-565	GAGCTCGATTCGATCCTATTAGAGACGTTCATTAATTTCATCATTGC
GTO-569	TCCACCAATCTGTTCTCTGTGAGC
GTO-570	TAATAGGATCGAATCGAGCTCCGTCGACAAGC
GTO-815	GTCTTTCCCGGGTCATCGCTATCGCACGTTCCAGATGTG
GTO-816	CAGGGCTGCAGATACCTACGAGGAAGGAAGTAACCAGC
GTO-844	TTCGCGATACTGTTGGAGCTCGCTG
GTO-845	GCTTTCTTGGTTGGCAAGCGCAAGTGTG
GTO-848	GCCTTCCATTCATCGCAAAGTTGGTCGAGCTCATCTTTTTCGGGAG
GTO-849	CCGTCTCAGCGAGCTCCAACAGTATCGCGAAGAGTTCTTGGTTGGC
GTO-850	CGAGCCTGTTGGAGCTCGCTGAGACG
GTO-851	CGAAAAAGCTTTGGTTGGCAAGCGC
GTO-852	GGCGGACTCGGTATGAATAGCGAGG
GTO-853	CGCCGCAATCAAATCATCTCCATCATG
GTO-854	ATGCGCAATCAAATCATCTCCATCATG
GTO-855	CGGACAATCGAATCCAAATTCCACC
GTO-906	GCAATCCACATGACGCCACCATCG
GTO-907	GGCGTAATACGACTCACTATAGGG
GTO-1362	CACCATGCCAAAAATTACACAAGATAGAATGG5
GTO-1376	GAAGCAACCCAACTAGCGCACATACAG
GTO-1377	GAGGTTGTGTGTTCACAGGTAGGAGG
O.10A	ACAGAGAACAGATTGGTGGATTATCTCCCAGTTCCTCTCCC
Ter-1-fw	AACCAGGGATTCAGTGCAGCTATCTTG
Ter-1-rev	CAAGATAGCTGCACTGAATCCCTGGTT
Tf-2-fw	AGCAATACTACACTACGCTATAATACACTACGTTGAGTATCACTATATGT
Tf-2-rev	ACATATAGTGATACTCAACGTAGTGTATTATAGCGTAGTGTAGTATTGCT
O.109	GAGCTCGATTCGATCCTATTAGAATTTTTCGCGATACTGTTGG
gGT4	CGAATCCAAA TTCCACCTAT AAATTATTCA AGACGGATCC<br>CCTATTTTCT ATGGAAGCTC<br>CCAAGATGGA ACTGAAGAGC TATAAACGAA AGAATGCTTC<br>GTTATCTCCC AGTTCCTCTC<br>CCGCCAAGGC ACAGCGTACT CACTTGTCCG CTGAGGAAAA<br>AATCAAGCTC ATGCGCTTAG<br>TCGTGCGTCA CAAGCACGAA CTCGTCGACC GTAAAACTAG<br>TGAGTTTTAC GCCAAGATCG<br>CCCGTATCGG TTATGAGGAC GAGGGCCTCG CTATTCATAC<br>CGAGTCCGCC TGTCGCAATC<br>AAATCATCTC CATCATGCGC GTCTACGAAC AGCGTTTGGC<br>CCATCGTCAA CCCGGCATGA<br>AGACCACTCC CGAAGAGGAT GAGGCTGACC AACTTTGCGA<br>TGAATGGAAG GCCCGTCTCA<br>GCGAGCTCCA ACAGTATCGC GAAAAATTCT TGGTTGGCAA<br>GCGCAAGTGT GATTGC

References

1. Arcangioli, B., Ghazvini, M. & Ribes, V. Identification of the DNA-binding domains of the switch-activating-protein Sap1 from S.pombe by random point mutations screening in E.coli. *Nucleic Acids Res.* **22,** 2930–2937 (1994).
